# Neuroimaging-based brain-age prediction in diverse forms of epilepsy: a signature of psychosis and beyond

**DOI:** 10.1038/s41380-019-0446-9

**Published:** 2019-06-03

**Authors:** Daichi Sone, Iman Beheshti, Norihide Maikusa, Miho Ota, Yukio Kimura, Noriko Sato, Matthias Koepp, Hiroshi Matsuda

**Affiliations:** 1 Integrative Brain Imaging Center, National Center of Neurology and Psychiatry, Tokyo, Japan; 2 Department of Clinical and Experimental Epilepsy, UCL Institute of Neurology, London, UK; 3 Department of Neuropsychiatry, Division of Clinical Medicine, Faculty of Medicine, University of Tsukuba, Ibaraki, Japan; 4 Department of Radiology, National Center of Neurology and Psychiatry, Tokyo, Japan

**Keywords:** Biological techniques, Neuroscience

## Abstract

Epilepsy is a diverse brain disorder, and the pathophysiology of its various forms and comorbidities is largely unknown. A recent machine learning method enables us to estimate an individual’s “brain-age” from MRI; this brain-age prediction is expected as a novel individual biomarker of neuropsychiatric disorders. The aims of this study were to estimate the brain-age for various categories of epilepsy and to evaluate clinical discrimination by brain-age for (1) the effect of psychosis on temporal lobe epilepsy (TLE), (2) psychogenic nonepileptic seizures (PNESs) from MRI-negative epilepsies, and (3) progressive myoclonic epilepsy (PME) from juvenile myoclonic epilepsy (JME). In total, 1196 T1-weighted MRI scans from healthy controls (HCs) were used to build a brain-age prediction model with support vector regression. Using the model, we calculated the brain-predicted age difference (brain-PAD: predicted age—chronological age) of the HCs and 318 patients with epilepsy. We compared the brain-PAD values based on the research questions. As a result, all categories of patients except for extra-temporal lobe focal epilepsy showed a significant increase in brain-PAD. TLE with hippocampal sclerosis presented a significantly higher brain-PAD than several other categories. The mean brain-PAD in TLE with inter-ictal psychosis was 10.9 years, which was significantly higher than TLE without psychosis (5.3 years). PNES showed a comparable mean brain-PAD (10.6 years) to that of epilepsy patients. PME had a higher brain-PAD than JME (22.0 vs. 9.3 years). In conclusion, neuroimaging-based brain-age prediction can provide novel insight into or clinical usefulness for the diverse symptoms of epilepsy.

## Introduction

Epilepsy is a common but quite diverse brain disorder [1]. Attempts at clinical classification of the disease are continuing, considering the many types of seizures, electroencephalogram (EEG) findings, structural abnormalities, and genetics [2]. In addition, although epileptic seizure is the main symptom of epilepsy, various forms of comorbidities often eventually occur in patients with epilepsy [3]. In particular, psychiatric and behavioral problems, including psychogenic nonepileptic seizures (PNESs), are one of the significant aspects of the condition [4]. However, the pathophysiology of these diverse forms of epilepsy and its related disorders is still unclear and needs to be elucidated for better clinical treatments.

On the other hand, machine learning in brain images has recently become expected to be used as a potential individual-level biomarker in many neuropsychiatric disorders [5]. There are already useful applications for automatic categorization of epilepsy neuroimaging [6]. In addition, more recent advances in machine learning have allowed us to predict the age of an individual’s brain image using regression models [7]. This “neuroimaging-based brain-age prediction” has been applied to several psychiatric disorders and Alzheimer’s disease [7, 8]. As for epilepsy, Pardoe et al. reported a significant 4.5-year increase in brain age compared with chronological age in refractory focal epilepsy [9]. We considered that the brain-age prediction could provide useful information on other forms of epilepsy. Thus, the initial aim of this study was to estimate brain age in various categories of epilepsy in accordance with the following three clinical research questions.

First is the effect of psychosis in epilepsy. Psychosis, which is an important comorbidity of epilepsy, is found in around 10‒20% of patients with temporal lobe epilepsy (TLE), which is higher than in the general population [10]. On the other hand, many other TLE patients do not develop psychosis, and no plausible explanation has yet been proposed for these different phenotypes. According to recent studies of brain age in schizophrenia [8, 11], patients with schizophrenia show a 3- to 5-year higher brain age compared with chronological age, and this gap accelerates around the onset of psychosis. Thus, we hypothesized that brain age could also be a novel biomarker in psychosis of epilepsy and possibly show higher values than in nonpsychotic patients.

Second, we aimed to investigate the differences between PNES and epilepsy with no lesion on visual assessment of MRI (i.e., magnetic resonance imaging (MRI)-negative epilepsy). PNES is defined as episodes resembling epileptic seizures but caused by psychogenic mechanisms [12]. Because PNES does not have epileptic physiology, patients with PNES should be treated differently from patients with epilepsy. Nevertheless, the diagnosis of PNES is sometimes difficult, especially with limited access to video-EEG monitoring as the gold-standard diagnosis of PNES confirming ictal semiology and EEG findings [12]. Actually, PNES is recognized as a diagnostic and therapeutic problem in many countries [13]. We considered that an MRI-based biomarker that could identify significant differences between PNES and epilepsy, particularly in visually normal MRI cases, would be helpful for clinicians and patients. Moreover, the results may shed additional light on the pathophysiology of PNES, although PNES is heterogeneous and cannot be explained by any single mechanism [14].

Third, we addressed the differences between progressive myoclonic epilepsy (PME) and juvenile myoclonic epilepsy (JME). PME is a group of neurodegenerative disorders characterized by myoclonic seizures and progressive neurological impairment, including ataxia or intellectual deterioration [15]. Because PME is often difficult to clinically distinguish from JME at the early stage, which is a more common and basically nonprogressive group with much better prognosis, some attempts have been made to demonstrate specific differences between PME and JME [16]. As with the second aim, we also applied brain-age prediction to this differentiation.

## Materials and Methods

### Healthy controls

To build and estimate the brain-age model, we recruited 1196 MRI scans at our center from healthy controls (HCs) with no history of neurological or psychiatric diseases and no use of medication affecting the central nervous system. No possible structural anomalies or abnormalities affecting the analysis were visually found in the controls on MRI. The 1196 HCs were aged between 20 and 89 years (mean ± SD: 55.4 ± 15.3 years) and comprised 426 men and 770 women. The mean ages and proportions of sex were different between each group of patients, but the HC database contained sufficient samples of each age and sex. Because epilepsy can affect individuals of all ages, we decided to include all available samples to establish a reliable brain-age model.

### Patients

Of the 437 adult (i.e., ≥20-year-old) patients with epilepsy or PNES recruited at our institute between November 2013 and December 2017, we enrolled 318 patients in the brain age analyses based on the following examinations and criteria.

All of the patients underwent careful clinical diagnosis by board-certified clinical epileptologists based on seizure semiology and conventional scalp EEG and conventional 3.0-T MRI inspection by experienced neuroradiologists. More detailed inclusion criteria and specific examinations performed for each category of epilepsy are described in Table 1.

**Table 1:** Enrollment criteria and detailed examinations and diagnoses in the initial six patient categories

Groups	*N*	Criteria	Visual MRI	FDG-PET performed in	Video-EEG performed in	Detailed/additional diagnoses
TLE-NL	164	- Presence of focal seizures consistent with TLE - Focal epileptiform discharges predominantly in temporal areas on routine EEG - Visually no abnormality on MRI	No abnormality in all participants	85 participants	43 participants	37 RTLE, 106 LTLE 21 unspecified/bilateral
TLE-HS	63	- Presence of focal seizures consistent with TLE - Focal epileptiform discharges predominantly in temporal areas on routine EEG - Visually unilateral HS on MRI	Unilateral HS in all participants	41 participants	25 participants	20 RTLE, 43 LTLE
Ext-FE	45	- Presence of focal epileptic seizures consistent with extra-temporal origin - Extra-temporal focal epileptiform discharges compatible with the seizures	FCD in 8 participants, and none in the others	33 participants	14 participants	19 FLE, 3 PLE, 2 OLE 21 multilobar/unknown origin
IGE	30	- Presence of primarily generalized seizures with no focal symptoms - Diffuse (poly)spike-wave complex on routine EEG - visually no abnormality on MRI	No abnormality in all participants	None	None	9 JME, 4 JAE, 16 IGE-GTC, 1 CAE
SGE/PME	5	- Presence of primarily generalized seizures - diffuse (poly)spike-wave complex on routine EEG - Clinical diagnosis of PME or epileptic encephalopathy - Visually no abnormality on MRI (except for unspecific mild atrophy)	No abnormality (except unspecific mild atrophy)	All participants	3 participants	4 PME (1 DRPLA), 1 SGE
PNES	11	- Presence of PNES confirmed on video-EEG monitoring - No epileptiform discharges nor epileptic seizures on video-EEG monitoring - Visually no abnormality on MRI	No abnormality in all participants	9 participants	All participants	2 PTSD 2 mild-intellectual disability

*TLE-NL* temporal lobe epilepsy with visually normal MRI, *TLE-HS* temporal lobe epilepsy with hippocampal sclerosis, *Ext-FE* extra-temporal lobe focal epilepsy, *IGE* idiopathic generalized epilepsy, *SGE/PME* symptomatic generalized epilepsy or progressive myoclonus epilepsy, *PNES* psychogenic nonepileptic seizures, *FCD* focal cortical dysplasia, *RTLE* right temporal lobe epilepsy, *LTLE* left temporal lobe epilepsy, *FLE* frontal lobe epilepsy, *PLE* parietal lobe epilepsy, *OLE* occipital lobe epilepsy, *JME* juvenile myoclonic epilepsy, *JAE* juvenile absence epilepsy, *IGE-GTC* idiopathic generalized epilepsy with generalized tonic-clonic seizures, *CAE* childhood absence epilepsy, *DRPLA* dentatorubral-pallidoluysian atrophy (genetically confirmed), *PTSD* post-traumatic stress disorder

The initial categorization of epilepsy at this stage was as follows: (1) TLE with no visible lesion (i.e., visually normal) on MRI (TLE-NL), (2) TLE with hippocampal sclerosis (TLE-HS), (3) extra-temporal lobe focal epilepsy (Ext-FE), (4) idiopathic generalized epilepsy (IGE), (5) PME or symptomatic generalized epilepsy (PME/SGE), and (6) PNES without any epileptic seizures (PNES).

The secondary categorization included psychosis vs. nonpsychosis in TLE, PNES vs. MRI-negative epilepsies, and JME vs. PME. The composition of MRI-negative epilepsies is shown in Supplementary Table 1. JME was diagnosed based on the presence of myoclonic seizures in addition to the criteria for IGE listed in Table 1.

The following exclusion criteria were applied to all patients: (1) a significant medical history of acute encephalitis, meningitis, severe head trauma, ischemic encephalopathy, or brain surgery; and (2) suspicious epileptogenic lesions (e.g., tumor, vascular malformation, and destructive lesion) on MRI other than unilateral HS or focal cortical dysplasia (FCD).

In total, 119 of the initial 437 patients were not enrolled, including 43 with focal epileptogenic lesions other than HS or FCD, 37 who were not classified into any specific categories, 11 with a history of encephalitis, 8 with severe head trauma, and 20 with other reasons (e.g., postneurosurgery and poor imaging quality).

All participants gave written informed consent. The study was approved by the Institutional Review Board at the National Center of Neurology and Psychiatry Hospital.

### Psychosis evaluation

We assessed the existence of inter-ictal psychosis (IIP) only in patients with TLE. Because TLE has the highest prevalence of psychosis, this investigation of psychosis was originally planned for TLE patients. The presence or history of IIP was diagnosed based on the Diagnostic and Statistical Manual of Mental Disorders, 4th edition criteria [17]. Of the 227 patients with TLE, 21 were diagnosed with IIP; the others had no psychotic episodes.

### MRI acquisition

The three-dimensional (3D) sagittal T1-weighted images of the HCs were obtained from two different protocols on 3.0-T MRI scanners: 798 individuals underwent Protocol 1 and the other 398 individuals underwent Protocol 2. On the other hand, all of the patients underwent Protocol 1.

Protocol 1: 3.0-T MR system (Philips Medical Systems, Best, The Netherlands) with the following protocol: repetition time (TR)/echo time (TE), 7.18 ms/3.46 ms; flip angle, 10°; number of excitations (NEX), 1; 0.68 × 0.68 mm^2^ in plane resolution; 0.6-mm effective slice thickness with no gap; 300 slices; matrix, 384 × 384; field of view (FOV), 26.1 × 26.1 cm.

Protocol 2: 3.0-T MR system (Verio, Siemens, Erlangen, Germany) with the following protocol: TR/TE, 1800 ms/2.25 ms; flip angle, 9°; NEX, 1; 0.87 × 0.78 mm^2^ in plane resolution; 0.8-mm effective slice thickness with no gap; 224 slices; matrix, 320 × 280; FOV, 25 × 25 cm.

### Neuroimaging processing

The pipeline of processing is described in Supplementary Fig. 1. Using SPM12 (Wellcome Trust Centre for Neuroimaging, London, UK; www.fl.ion.ucl.ac.uk/spm/), all 3D T1-weighted images were bias-corrected and segmented into gray matter (GM), white matter (WM), and cerebrospinal fluid components. The GM and WM components were used. To ensure the accuracy of the image segmentation, all segmented GM and WM images were visually inspected. We then used SPM DARTEL [18] to conduct a nonlinear registration to a custom template on the basis of a training dataset (i.e., healthy individuals, [*N* = 1196]). The imaged GM and WM were then registered to MNI space, modulated to retain tissue volume information, and smoothed with a 4-mm Gaussian kernel [19, 20]. As per the pipeline proposed in [21], the spatially normalized GM and WM images were resampled into 8 mm isotropic spatial resolution. For each individual, the voxel intensities extracted from smoothed GM and WM images were concatenated to build a whole-brain estimating age and considered as raw features for the regression model.

### Regression model and validation

To explore the brain age in various forms of epilepsy, we used a standard nu-support vector regression (nu-SVR) model conducted in LIBSVM (http://www.csie.ntu.edu.tw/~cjlin/libsvm/) toolbox with linear kernel and default set of parameters (i.e., in the LIBSVM: *C* = 1, *v* = 0.5). SVR has previously shown a robust performance in estimating brain age from T1-weighted MRI images [22]. Following [21], a principal component analysis was used to reduce the probability of overfitting and overcome the curse of dimensionality. The number of principal components was set at 100 per individual.

Consequently, for the regression model, the chronological age was considered the dependent variable and the principal components derived from the concatenated GM and WM voxel intensities were considered the independent variables. To assess the ability of the proposed regression model, we conducted a tenfold cross validation on the training set (i.e., healthy individuals), with onefold in each iteration considered as the test and the remaining folds considered the training set. The model accuracy was measured via the mean absolute error, root mean squared error, and the correlation between the chronological age and estimated age through tenfold cross validation. Thereafter, the final regression model was built using the entire training set (i.e., healthy individuals [*N* = 1196]) and then applied to epilepsy patients (*N* = 318) to estimate the brain ages.

### Group comparisons and correlations of the brain-predicted age difference

Based on the age predicted by the MRI-based SVR model, we calculated each participant’s brain-predicted age difference (brain-PAD: predicted age—chronological age). First, we compared the mean brain-PAD among the six initial categories of patients and the HCs. In addition, correlations of the brain-PAD with disease duration or onset age were investigated in each initial category. For the three clinical differentiations, we evaluated the relevant two groups by comparing the mean brain-PAD and performing receiver operating characteristic (ROC) curve and area under the curve (AUC) analyses. However, we refrained from using inferential statistics for the comparison between JME and PME because of the small sample sizes of the two groups.

### Statistics

Statistical analyses were performed by SPSS software, version 23.0 (SPSS Japan, Tokyo). The mean brain-PAD was compared via analysis of covariance (ANCOVA) with age and sex as covariates. Because onset age or disease duration in each group did not show a normal distribution, the correlations of the brain-PAD with these parameters were analyzed by a nonparametric method (i.e., Spearman’s rank correlation coefficient) with Bonferroni correction for multiple comparisons. The ROC curves were nonparametrically analyzed according to whether the AUC was significantly higher than 0.5 (i.e., random) to differentiate the two relevant clinical categories. Other clinical parameters than brain-PAD were compared by an unpaired *t* test for continuous variables and a Pearson’s *χ*^2^ test for binary parameters. A *p* value < 0.05 was deemed significant.

This study included various types of analysis, and then not all analyses had the sample sizes validated. However, in the comparisons among initial categories, psychosis vs. nonpsychosis, and PNES vs. MRI-negative epilepsies, the total sample sizes were demonstrated to achieve 80% power to detect differences with 0.25 effect sizes based on G*Power 3.1.9.4 [23].

## Results

### Clinical demographics

The clinical demographics are presented in Table 2. Each group showed differing distributions of age, sex, and disease onset/duration. Most patients had refractory seizures except those in the IGE group.

**Table 2:** Clinical demographics and brain-PAD results in the initial categorization of patients

	TLE-NL (*N* = 164)	TLE-HS (*N* = 63)	Ext-FE (*N* = 45)	IGE (*N* = 30)	SGE/PME (*N* = 5)	PNES (*N* = 11)
Age (y)						
Mean ± SD	45.8 ± 16.6	43.3 ± 13.7	35.9 ± 12.0	28.9 ± 7.7	31.4 ± 9.8	31.5 ± 8.6
Sex (*N*)						
Male:female	81:83	25:38	27:18	8:22	3:2	3:8
Disease duration (y)						
Mean onset age ± SD	30.8 ± 20.8	14.4 ± 10.9	13.3 ± 10.5	15.6 ± 6.1	10.6 ± 8.7	23.6 ± 11.1
Mean duration ± SD	15.0 ± 13.9	29.0 ± 13.5	22.6 ± 12.6	13.3 ± 9.3	20.8 ± 15.7	7.8 ± 7.3
Drug-resistance (*N*, %)						
Patients with refractory SZ	149 (91%)	61 (97%)	43 (96%)	6 (20%)	5 (100%)	11 (100%)
Brain-age estimation (y)						
Mean brain-PAD ± SD	4.7 ± 7.9	8.8 ± 7.3	5.6 ± 7.5	8.9 ± 6.3	21.2 ± 10.0	10.6 ± 5.6
Estimated marginal mean ± SE	3.3 ± 0.5	6.9 ± 0.8	2.1 ± 0.9	3.8 ± 1.1	16.7 ± 2.7	6.1 ± 1.8

*TLE-NL* temporal lobe epilepsy with visually normal MRI, *TLE-HS* temporal lobe epilepsy with hippocampal sclerosis, *Ext-FE* extra-temporal lobe focal epilepsy, *IGE* idiopathic generalized epilepsy, *SGE/PME* symptomatic generalized epilepsy or progressive myoclonic epilepsy, *PNES* psychogenic nonepileptic seizures, *SZ* seizures, *brain-PAD* brain-predicted age difference, *SE* standard error

### Brain-age prediction model in HCs

**Fig. 1:**
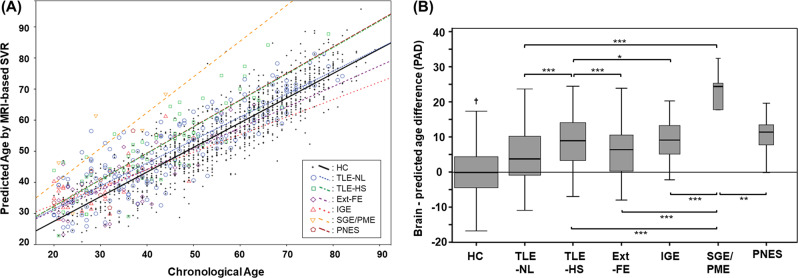
**a** The scatter plots and linear fits of the individual predicted brain and chronological ages in each group. **b** The box plot of brain-PAD in each group. *p < 0.05, **p < 0.01, ***p < 0.001, ^†^HCs showed significantly lower brain-PAD than TLE-NL (*p* < 0.001), TLE-HS (*p* < 0.001), IGE (*p* < 0.01), SGE/PME (*p* < 0.001), and PNES (*p* < 0.01), but the difference was not significant for Ext-FE (*p* = 0.134). The *p* values are corrected by ANCOVA. The width of the boxes is weighted by the logarithm of the number of subjects in each group

Figure 1 contains each individual’s predicted age and chronological age. The SVR brain-age prediction model showed a mean absolute error of 5.28 years in HCs, and the predicted age in HCs was highly correlated with their chronological age (*r* = 0.90). The mean (±SD) brain-PAD in HCs was 0.13 (±6.7) years.

Because there are different age and sex distributions between the two MRI scanners (Supplementary Fig. 2a), we investigated the scanner effect for brain-PAD. Consequently, as in Supplementary Fig. 2b, the two scanners showed a similar pattern for brain-PAD among their HCs with no significant difference in the mean value (*p* = 0.299, unpaired *t* test).

### Brain-PAD in the six categories of patients

As per the initial aim of this study, we evaluated the brain-PAD of the initial categorizations of patients. All six categories of patients showed a higher mean brain-PAD compared with HCs, and the differences were statistically significant for five of the groups, with the exception of Ext-FE (*p* = 0.149) (Table 2 and Fig. 1). The PME/SGE group had the highest brain-PAD and the TLE-HS group showed a significantly higher mean brain-PAD than the TLE-NL group. The brain-PAD values corrected for age and sex (i.e., estimated marginal means by ANCOVA) are also shown in Table 2 and Supplementary Fig. 3a.

In addition, the onset age in TLE-NL was negatively correlated with the brain-PAD (Spearman’s *r*s = −0.436, *p* < 0.001, Supplementary Fig. 3b). All other correlations were insignificant.

### Psychosis vs. nonpsychosis in TLE

**Fig. 2:**
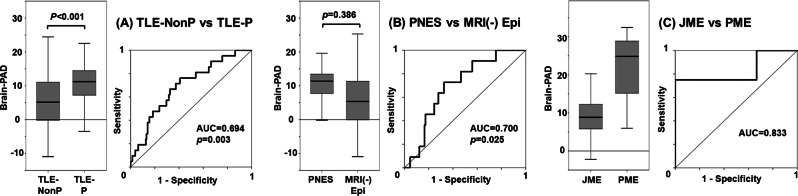
The comparisons of the brain-PAD and discrimination of each clinical categorization. **a** TLE without psychosis (TLE-Non-P) vs. TLE with psychosis (TLE-P), **b** PNES vs. all MRI-negative epilepsies (MRI(−) Epi), and **c** JME vs. PME

The clinical demographics and brain-PAD comparison results are shown in Table 3 and Fig. 2. The TLE with psychosis group showed a significantly higher brain-PAD than the TLE without psychosis group (*p* < 0.001, ANCOVA). Because TLE with HS has higher brain-PAD, we added the existence of HS as covariate post hoc, but the significance remained (*p* = 0.005). There is no significant interaction for brain-PAD between the existence of HS and psychosis (*p* = 0.898). The detailed demographics of these categorizations are presented in Supplementary Table 2. The AUC was 0.694 for differentiating psychotic from nonpsychotic patients in TLE.

**Table 3:** Clinical demographics and brain-PAD results for each comparison

	Psychosis vs. nonpsychosis in TLE			PNES vs. MRI-negative epilepsies			JME vs. PME		
	TLE-Non-P (*N* = 206)	TLE-P (*N* = 21)	*p*	PNES (*N* = 11)	MRI(−) Epi (*N* = 236)	*p*	JME (*N* = 9)	PME (*N* = 4)	*p*
Age (y)									
Mean ± SD	45.1 ± 16.3	45.0 ± 11.2	0.976	31.5 ± 8.6	41.6 ± 16.3	0.003	24.1 ± 4.6	29.5 ± 10.2	0.202
Sex (*N*)									
Male:female	98:108	8:13	0.407	3:8	114:122	0.172	1:8	2:2	0.125
Disease duration (y)									
Mean onset age ± SD	27.6 ± 20.2	12.3 ± 9.8	<0.001	23.6 ± 11.1	25.9 ± 19.5	0.537	15.1 ± 3.5	11.3 ± 9.9	0.500
Mean duration ± SD	17.4 ± 14.7	32.7 ± 11.5	<0.001	7.8 ± 7.3	15.7 ± 13.2	0.005	9.0 ± 5.3	18.3 ± 16.8	0.355
Drug-resistance (*N*)									
Patients with refractory SZ	190	20	0.618	11	195	0.130	2	4	0.009
Other									
Patients with HS (*N*)	50	13	<0.001	NA	NA	NA	NA	NA	NA
Onset age of psychosis (y)	NA	27.2 ± 13.1	NA	NA	NA	NA	NA	NA	NA
Brain-age estimation (y)									
Mean brain-PAD ± SD	5.3 ± 7.7	10.9 ± 7.8	<0.001	10.6 ± 5.6	5.8 ± 8.1	0.386	9.3 ± 6.6	22.0 ± 11.3	NA

*brain-PAD* brain-predicted age difference, *TLE-Non-P* temporal lobe epilepsy without psychosis, *TLE-P* temporal lobe epilepsy with psychosis, *SZ* seizures, *MRI(−) Epi* MRI-negative epilepsies, *HS* hippocampal sclerosis

Comparisons of the mean brain-PAD were analyzed by ANCOVA. All other *p* values were generated by an unpaired *t* test or Pearson’s *χ*^2^ test

Statistical comparison of the mean brain-PAD between JME and PME was not performed in consideration of the small sample size

### PNES vs. MRI-negative epilepsies

This section compared 11 patients with PNES and 236 patients with MRI-negative epilepsies, including 164 TLE-NL, 37 MRI-negative cases from the Ext-FE group, 30 IGE, and 5 PME/SGE. The mean brain-PAD was not significantly different between PNES and MRI-negative epilepsies by ANCOVA comparison, although the PNES group had higher brain-PAD values (Table 3 and Fig. 2). On the other hand, the ROC curve of differentiation by the raw brain-PAD values showed an AUC of 0.700.

### JME vs. PME

We enrolled nine JME patients and four PME patients from the IGE and PME/SGE categories, respectively. The mean brain-PAD was higher in PME than in JME (Table 3 and Fig. 2).

## Discussion

In this study, we performed neuroimaging-based brain-age prediction for various forms of epilepsy. The brain age in HCs was consistent across MRI scanners, which might suggest that the algorithm for brain age can provide a good biomarker beyond scanner differences. As the initial result, all categories of patients showed significantly increased brain-age values, with only Ext-FE showing a statistical trend level value. In the six categories, SGE/PME, which is the most severe phenotype, showed the highest brain-PAD, and TLE-HS, which is accompanied by distinct brain morphological changes, had higher values than other epilepsy syndromes.

A previous brain-age study of epilepsy showed a 4.5-year increase in brain-PAD in 94 patients with refractory epilepsy [9]; this previous study consisted of various focus locations and partly included HS and FCD. This value would be consistent with our results in TLE with normal MRI or extra-temporal epilepsy groups (Table 2). Although they also reported no significant effect of HS on brain-PAD in their 12 patients with TLE and HS, our study, involving a higher number of samples (*N* = 63), suggested the significant effect of HS on the increased brain age.

The previous study also reported a significantly negative correlation of brain-PAD with onset age and no significant association with duration of disease [9]. We also found the same correlation in the TLE-NL group (Supplementary Fig. 3b). This association between an increased brain-PAD and an earlier onset of epilepsy could suggest that the brain structural brain-age prediction may reflect the vulnerability to seizure generation or initial brain insult rather than disease progression. However, based on our results in each category, these correlations could depend on the type of epilepsy. In TLE-NL, conventional volumetry found no significant abnormality [24], despite the abnormal cortical thickness [25] and WM integrity [26]. Our voxel-based brain-age prediction may provide novel insights into structural neuroimaging in this group.

In addition, we evaluated the effect of psychosis on TLE. The mean brain-PAD in TLE with psychosis was 10.9 years, which was significantly higher than that in TLE without psychosis (5.3 years). Considering the reported 5.5-year increase in brain-PAD in schizophrenia [8], our result should be concordant with the comorbidity of psychosis and TLE. It might also suggest that the mechanism underlying the increased brain age in TLE would be different from that in psychosis. In fact, there is no solid agreement in brain volumetry in psychosis of epilepsy due to partly conflicting results of past studies [27] and there has recently been an attempt to detect network abnormalities beyond mere morphology [28]. Considering the importance of early detection and intervention for psychosis [29], more specific and individual-level biomarkers are desirable. The brain-age prediction was suggested as a potential biomarker of vulnerability to psychosis development [8] or accelerated neuromaturation [11]. Although a longitudinal survey is needed, we speculate that brain-age prediction could be a candidate biomarker of psychosis in epilepsy.

In addition, we focused on other types of epilepsy and PNES. Even the IGE patients with mostly controlled seizures showed higher values of the brain-PAD. Probably, the brain-PAD increase cannot simply be explained by refractory seizures and that, particularly in IGE, it might reflect the frontal lobe or thalamic dysfunction [30, 31]. Surprisingly, PNES patients with no electrophysiological seizures also showed a significantly increased brain-PAD, which was comparable with or even higher than that in MRI-negative epilepsies. A recent neuroimaging review suggested functional and structural alterations, particularly related to emotion processing and cognitive–executive control, as a neurobiological mechanism in PNES [32]. This unstable cognitive–emotional–attention system could be associated with increased aging of the brain in PNES. The pathophysiology of PNES is still unclear, and our study may provide further information on it.

Furthermore, we also demonstrated a much greater increase in brain-PAD in PME compared with JME. This differentiation is still a clinical problem [16], and our results appear to agree with the differences between the two diseases, given the severe phenotype of PME [15]. However, the sample size in this comparison was small and patients with PME had a certain level of disease duration. Therefore, the current results cannot yield clear evidence on the utility of the brain age for differentiating myoclonic epilepsy in the early stages, although we believe that we have preliminarily shown its potential usefulness.

Moreover, we should discuss the underlying mechanisms of the altered brain age in epilepsy. Given that epilepsy is characterized by abnormal excessive neuronal activity (i.e., seizures) [1], recurrent electric damage may accelerate the brain aging in epilepsy. This scenario could be more applicable to our SGE/PME group, which represents the most severe category with extremely refractory, often daily, seizures, and intellectual deterioration. However, even the drug-responsive group (i.e., IGE) showed a certain level of increase in brain age. One potential explanation would be underlying predispositions in epilepsy, which might have affected patients’ aging process throughout life including neurodevelopment. Another possible factor is inter-ictal epileptic discharges, which can be seen even in seizure-free cases, given the recent evidence of association with cognitive impairment [33]. In addition, recent evidence is revealing the progressive GM loss or abnormal tau deposition in epilepsy [34, 35]. Thus, our observation of increased brain aging is also consistent with a potentially neurodegenerative pathophysiology in epilepsy.

For clinical settings, brain age seems generally sensitive to the presence of epilepsy, but the within-group variability of brain-PAD is high. In addition, multiple nonepileptic conditions show an increased brain age [36]. Considering that the usefulness of brain age has been reported mostly for psychiatric disorders and cognitive impairment, brain age may have clinical potential for neurocognitive changes or psychiatric comorbidities in epilepsy. Furthermore, it is known that increased brain age predicts mortality risk [37]. Given that epilepsy is associated with an increased risk of sudden death [38], possibly related to specific brain structures essential for cardiorespiratory recovery [39], future application of brain age might be beneficial to identify patients at high risk of sudden death.

This study has several limitations. First, each group of patients had differing age/sex distributions, sample sizes, and diagnosis criteria, although we partly corrected for these differences using statistical methods. We should carefully interpret the current results, particularly those obtained in groups with a small sample size. Another limitation is the lack of psychiatric or psychological information other than psychosis, such as depression or intellectual disability. Given the high prevalence of intellectual disability in PNES [12], further investigations with neurocognitive data would be desirable, especially for PNES. Additionally, the cross-sectional design cannot answer questions about causal relationships or predictability in early stages. In particular, the usefulness for the differentiation of myoclonic epilepsy in the early stages is still unclear, considering the stage of our PME cohort. To address these limitations, further longitudinal validations with more detailed clinical information are needed.

In conclusion, we found increased brain-PAD in most types of epilepsy, with SGE/PME and TLE with HS showing particularly high values. In addition, TLE with psychosis presents a significant increase in brain-PAD compared with nonpsychotic TLE. Thus, brain-age prediction can provide a novel insight into or clinical usefulness for the diverse symptoms of epilepsy.

## Supplementary information

Supplementary Table 1

Supplementary Table 2

Supplementary Figure Legends

Supplementary Figure 1

Supplementary Figure 2

Supplementary Figure 3
