# Supplementary material for: Neuroimaging-based brain-age prediction in diverse forms of epilepsy: a signature of psychosis and beyond
Source: Mol Psychiatry. 2019 Jun 3;26(3):825–34. doi: 10.1038/s41380-019-0446-9 (PMC7910210; doi:10.1038/s41380-019-0446-9)
Supplement: Supplementary file 1 — Supplementary Table 1 [file 41380_2019_446_MOESM1_ESM.docx]

Supplementary Table 1. The composition and criteria for the secondary category of MRI-negative epilepsies.

| Group | N | Criteria | Visual MRI | Composition |
| --- | --- | --- | --- | --- |
| MRI-negative epilepsies | 236 | - presence of any kind of epileptic seizures  - presence of epileptiform discharges on EEG  - visually no evident abnormality on MRI | No evidence of abnormality in all participants | - All 164 TLE-NL - 37 Ext-FE (excluding 8 cases with FCD) - All 30 IGE - All 5 PME/SGE |
