# Supplementary material for: Neuroimaging-based brain-age prediction in diverse forms of epilepsy: a signature of psychosis and beyond
Source: Mol Psychiatry. 2019 Jun 3;26(3):825–34. doi: 10.1038/s41380-019-0446-9 (PMC7910210; doi:10.1038/s41380-019-0446-9)
Supplement: Supplementary file 2 — Supplementary Table 2 [file 41380_2019_446_MOESM2_ESM.docx]

|  |  | TLE-NL | TLE-HS | Total |
| --- | --- | --- | --- | --- |
| TLE-NonP | Number of Subjects | 156 | 50 | 206 |
|  | Mean age ± SD | 46.2 ± 16.8 | 41.2 ± 14.2 | 45.1 ± 16.3 |
|  | Male:Female | 77:79 | 21:29 | 98:108 |
|  | Mean onset age ± SD | 31.8 ± 20.7 | 14.6 ± 10.9 | 27.6 ± 20.2 |
|  | Mean duration ± SD | 14.3 ± 13.8 | 27.1 ± 13.2 | 17.4 ± 14.7 |
|  | Mean brain-PAD ± SD | 4.4 ± 7.7 | 8.2 ± 7.1 | 5.3 ± 7.7 |
| TLE-P | Number of Subjects | 8 | 13 | 21 |
|  | Mean age ± SD | 38.0 ± 10.5 | 49.3 ± 9.6 | 45.0 ± 11.2 |
|  | Male:Female | 4:4 | 4:9 | 8:13 |
|  | Mean onset age ± SD | 10.6 ± 6.9 | 13.3 ± 6.5 | 12.3 ± 9.8 |
|  | Mean duration ± SD | 27.4 ± 7.7 | 36.0 ± 12.4 | 32.7 ± 11.5 |
|  | Mean brain-PAD ± SD | 10.5 ± 8.5 | 11.2 ± 7.7 | 10.9 ± 7.8 |
| Total | Number of Subjects | 164 | 63 | 227 |
|  | Mean age ± SD | 45.8 ± 16.6 | 43.3 ± 13.7 | 45.1 ± 15.9 |
|  | Male:Female | 81:83 | 25:38 | 106:121 |
|  | Mean onset age ± SD | 30.8 ± 20.8 | 14.4 ± 10.9 | 26.2 ± 20.0 |
|  | Mean duration ± SD | 15.0 ± 13.9 | 29.0 ± 13.5 | 18.8 ± 15.1 |
|  | Mean brain-PAD ± SD | 4.7 ± 7.9 | 8.8 ± 7.3 | 5.9 ± 7.9 |

Supplementary Table 2. The detailed demographics of two categorizations of patients with TLE.
