# Supplementary material for: Neuroimaging-based brain-age prediction in diverse forms of epilepsy: a signature of psychosis and beyond
Source: Mol Psychiatry. 2019 Jun 3;26(3):825–34. doi: 10.1038/s41380-019-0446-9 (PMC7910210; doi:10.1038/s41380-019-0446-9)
Supplement: Supplementary file 3 — Supplementary Figure Legends [file 41380_2019_446_MOESM3_ESM.docx]

**Supplementary Figure Legends**

**Supplementary Figure 1.** The pipelines of the MRI processing and brain age model building in this study.

**Supplementary Figure 2.** (A) The age and sex distributions between the two MRI scanners. (B) The histogram distribution of brain-PAD in the training set with respect to scanner.

**Supplementary Figure 3.** (A) Corrected brain-PAD values for age and sex (i.e., estimated marginal means) in each group. Error bar denotes 2 SE (standard error). (B) A significant negative correlation was noted between brain-PAD and onset age in TLE with no visible lesion on MRI (TLE-NL group).
