# Supplementary figures and images for: Neuroimaging-based brain-age prediction in diverse forms of epilepsy: a signature of psychosis and beyond
Source: Mol Psychiatry. 2019 Jun 3;26(3):825–34. doi: 10.1038/s41380-019-0446-9 (PMC7910210; doi:10.1038/s41380-019-0446-9)

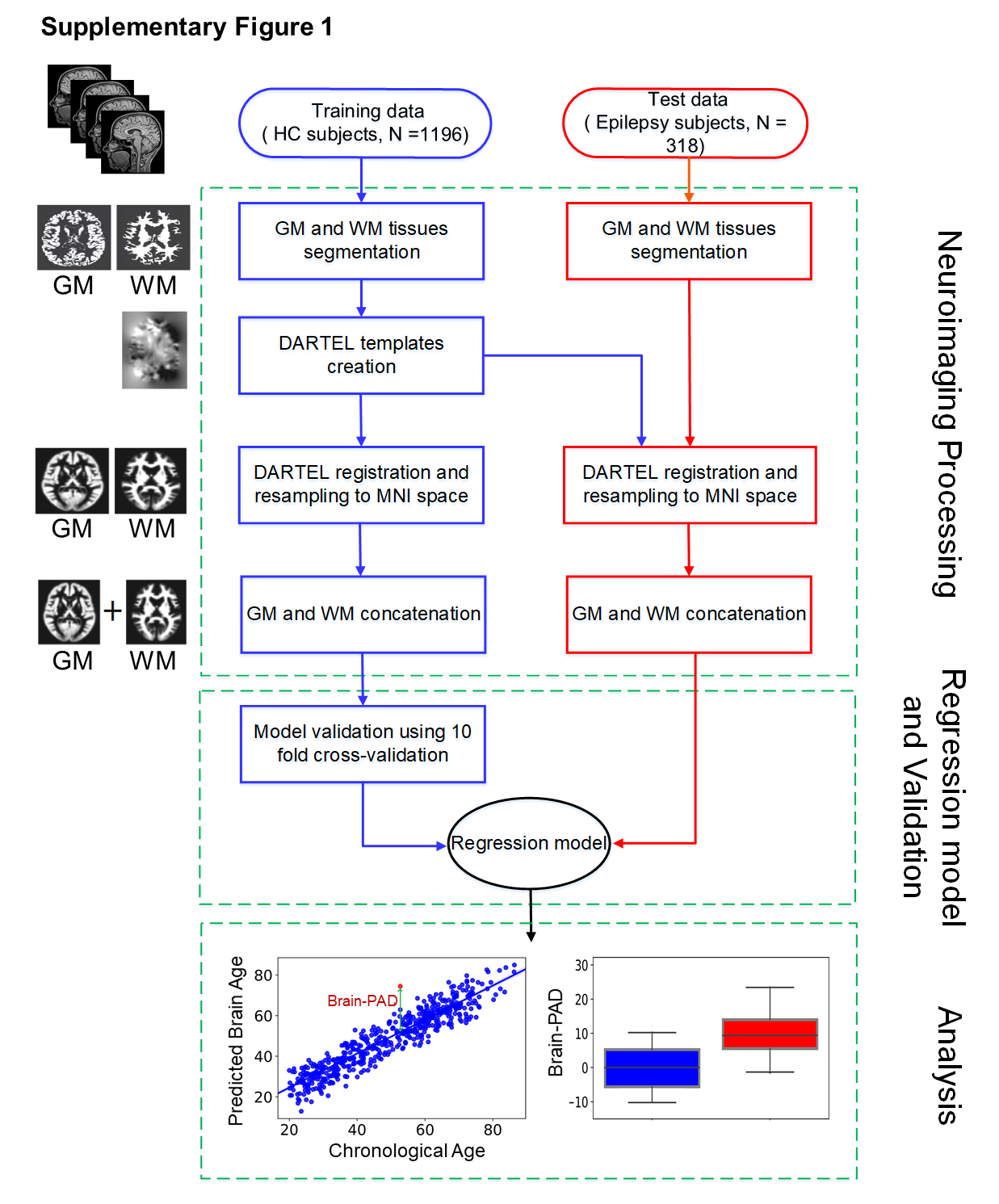

Supplement: Supplementary file 4 — Supplementary Figure 1 [file 41380_2019_446_MOESM4_ESM.tif]

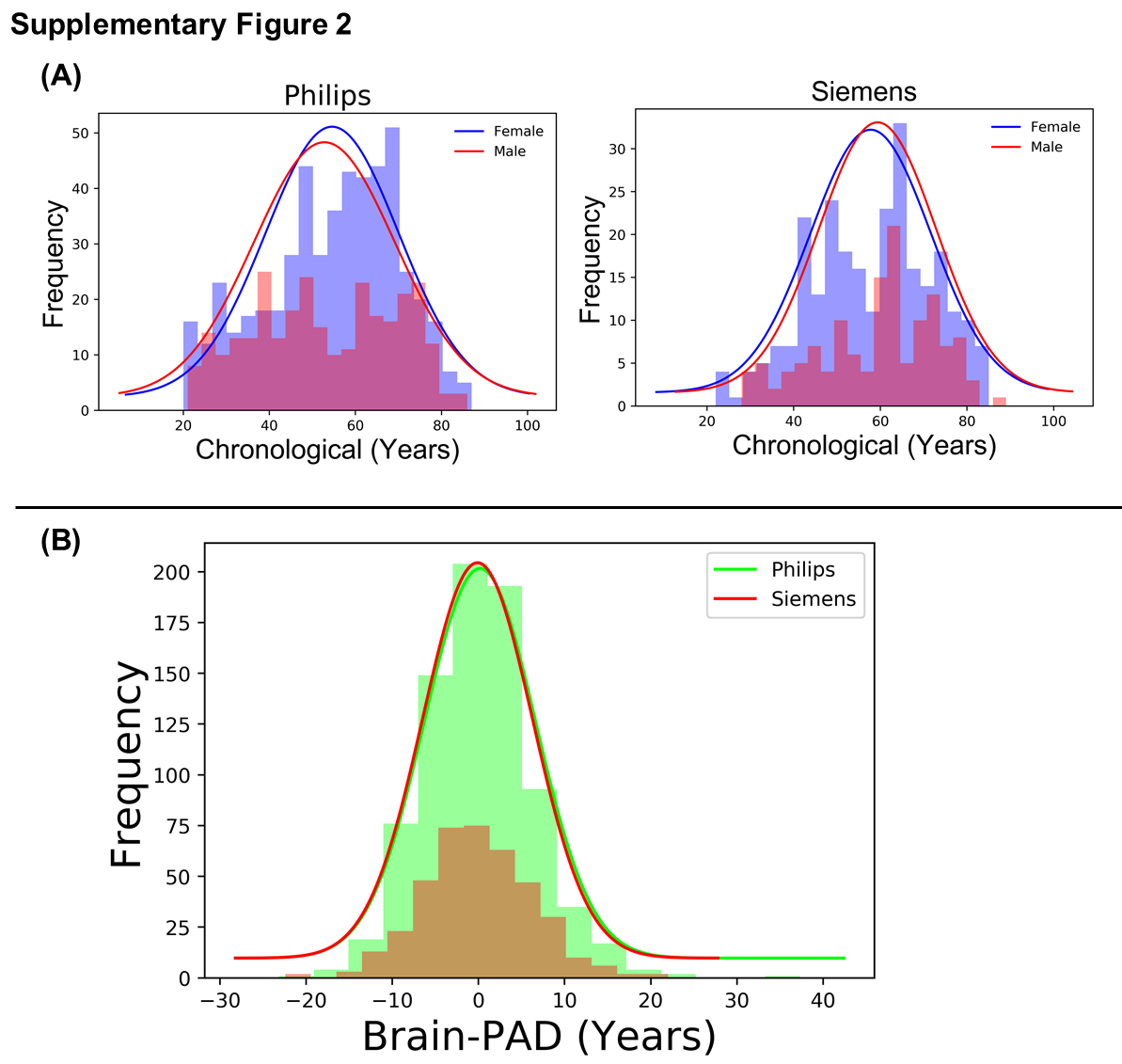

Supplement: Supplementary file 5 — Supplementary Figure 2 [file 41380_2019_446_MOESM5_ESM.tif]

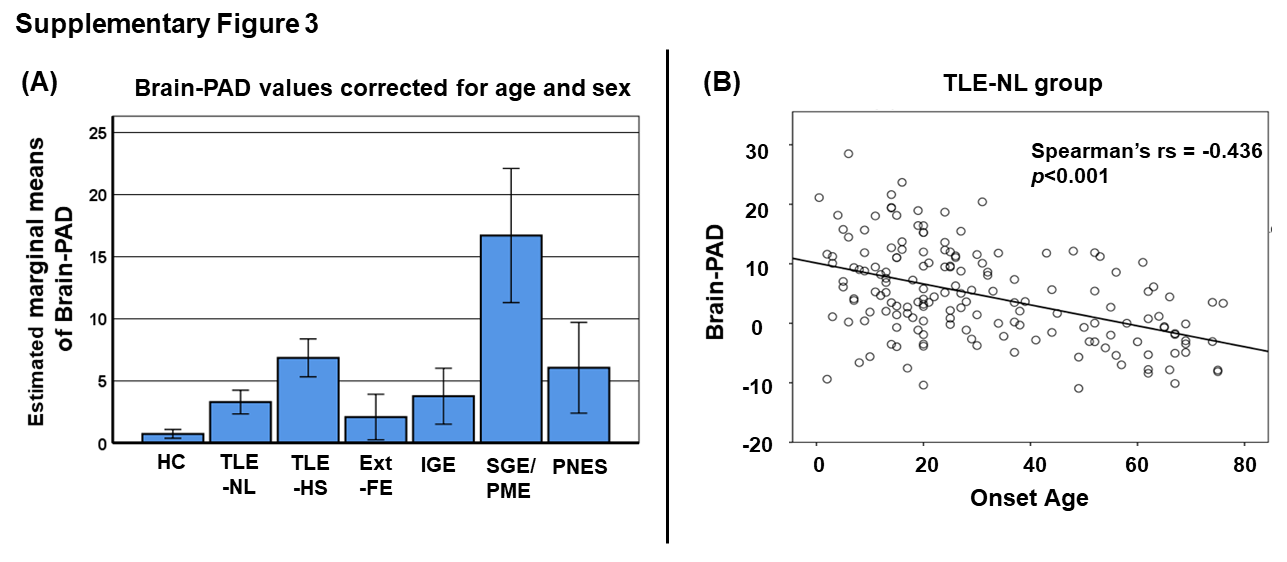

Supplement: Supplementary file 6 — Supplementary Figure 3 [file 41380_2019_446_MOESM6_ESM.tif]
